# Spatially Encoded Protocell Network for Non‐Cascaded Parallel Biocomputation

**DOI:** 10.1002/advs.77019

**Published:** 2026-08-18

**Authors:** Shuqi Wu, Liangfei Tian

**Affiliations:** ^1^ Zhejiang Key Laboratory of Intelligent Sensing Technology and Advanced Medical Instrument, Key Laboratory of Biomedical Engineering of Ministry of Education, College of Biomedical Engineering & Instrument Science Zhejiang University Hangzhou China; ^2^ Innovation Center for Smart Medical Technologies & Devices Binjiang Institute of Zhejiang University Hangzhou China; ^3^ Department of Ambulatory Surgery Women's Hospital School of Medicine Zhejiang University Hangzhou China

**Keywords:** biocomputation, full‐adder operation, half‐adder operation, protocell

## Abstract

Biological systems inspire computing paradigms by autonomously regulating material and information flows through molecular interactions. While biomolecules such as enzymes and DNA have been harnessed for biocomputation, implementing complex logic operations remains challenging due to signal attenuation, crosstalk, and instability inherent in molecular cascades. To overcome these limitations, we present a non‐cascaded biocomputing architecture based on spatially encoded, gradient‐responsive protocell communities embedded in a three‐dimensional hydrogel matrix. By leveraging differential reaction–diffusion dynamics of input signals, distinct output regions are established across the spatial domain, enabling parallel, multi‐threshold computation without signal routing. This architecture achieves continuous spatial encoding and allows simultaneous readout of all output regions, supporting nonlinear logic operations with high fault tolerance. Using this framework, we demonstrate the successful implementation of both half‐adder and full‐adder logic operations via two‐input/two‐output and three‐input/two‐output configurations, respectively. Our approach provides a scalable, programmable platform for parallel biocomputation, offering a promising route toward low‐complexity, robust, and non‐cascaded molecular computing systems that bridge synthetic biology and computational science.

## Graphical abstract

A non‐cascaded, hydrogel‐based proteinosome computational platform is developed, in which redox‐responsive proteinosomes serve as local computing units within a shared reaction–diffusion field. Reductant inputs, countered by an auxiliary glucose gradient, regulate spatially patterned proteinosome disruption, and generate population‐level boundaries between degraded and intact protocells as collective readouts that are discretized into binary output states.

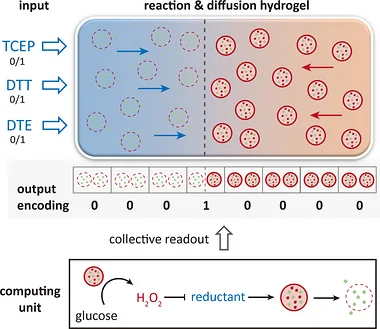

## 1 Introduction

Inspired by the autonomous regulation of material and information fluxes in living systems [1], biocomputing aims to transduce the dynamic reactivity of biomolecules and responsive entities—including enzymes, nucleic acids, engineered cells, and functional materials—into programmable output signals [2, 3]. This paradigm establishes a molecular‐level logic‐embedded architecture that holds transformative potential for biosensing [4, 5], programmed drug delivery [6, 7], and synthetic biology [8, 9]. Current biocomputing designs predominantly rely on electronic Boolean logic, constructing modular reaction networks to facilitate information processing [10, 11]. While cascaded strategies with DNA circuits and enzymatic cascades, have successfully implemented fundamental logic gates and arithmetic units [8, 12, 13, 14, 15, 16, 17, 18, 19, 20] by simplifying complex chemical responses into binary “0/1” outputs, they are inherently limited by significant signal propagation delays and cumulative attenuation due to their multi‐step sequential nature [21, 22]. In contrast, biological systems utilize non‐equilibrium reaction networks [23, 24, 25, 26, 27, 28, 29] and compartmentalized structures [30, 31, 32, 33, 34, 35, 36, 37, 38, 39, 40, 41] to amplify kinetic disparities during reaction [42, 43, 44, 45, 46, 47] or diffusion processes [48, 49, 50, 51, 52, 53, 54, 55, 56] and suppress stochastic noise [57, 58], thereby enabling high‐efficiency parallel information processing [59]. Therefore, a key challenge in biocomputing is to exploit intrinsic differences in biochemical kinetics to build high‐capacity parallel architectures without multi‐step cascade reactions for the efficient encoding and execution of complex logic in advanced biocomputing systems.

Herein, we report a non‐cascaded computing architecture based on the spatial encoding of environment‐responsive protocell populations driven by reaction–diffusion (RD) kinetics. Conceptually, this architecture relies on three interconnected elements, including chemically responsive units that convert local molecular environments into readable states, a shared reaction–diffusion field that integrates multiple inputs without sequential intermediate transfer, and predefined spatial readout domains that decode continuous response patterns into discrete output states. In this framework, chemical inputs are processed simultaneously within the same physicochemical medium rather than being relayed through cascaded reaction modules (Figure 1). This strategy exploits the intrinsic physicochemical disparities of input molecules to transform complex logic operations into parallelized spatial patterning, effectively circumventing the cumulative errors associated with temporal cascades. Specifically, we implemented this framework using redox‐responsive protocells uniformly embedded within a 3D hydrogel matrix. The protocells serve as local response units, while the hydrogel provides a shared RD field in which different chemical inputs propagate, interact, and generate input‐dependent spatial response profiles. By partitioning the hydrogel into predefined output domains, these continuous response profiles are converted into discrete spatial codes, thereby establishing the logic relationships between chemical inputs and output states. Based on this framework, we regulated the individual and combined RD kinetics of two input molecules to achieve spatial programming of a two‐input/two‐output half‐adder. Furthermore, by introducing bidirectional diffusion gradients, we demonstrated a distributed full‐adder where a single input combination generates two spatially separated response regions for parallel output‐state decoding. This spatial response platform provides a programmable paradigm for parallel non‐cascaded computing, offering a new trajectory for the development of advanced biocomputing logical devices.

**FIGURE 1:**
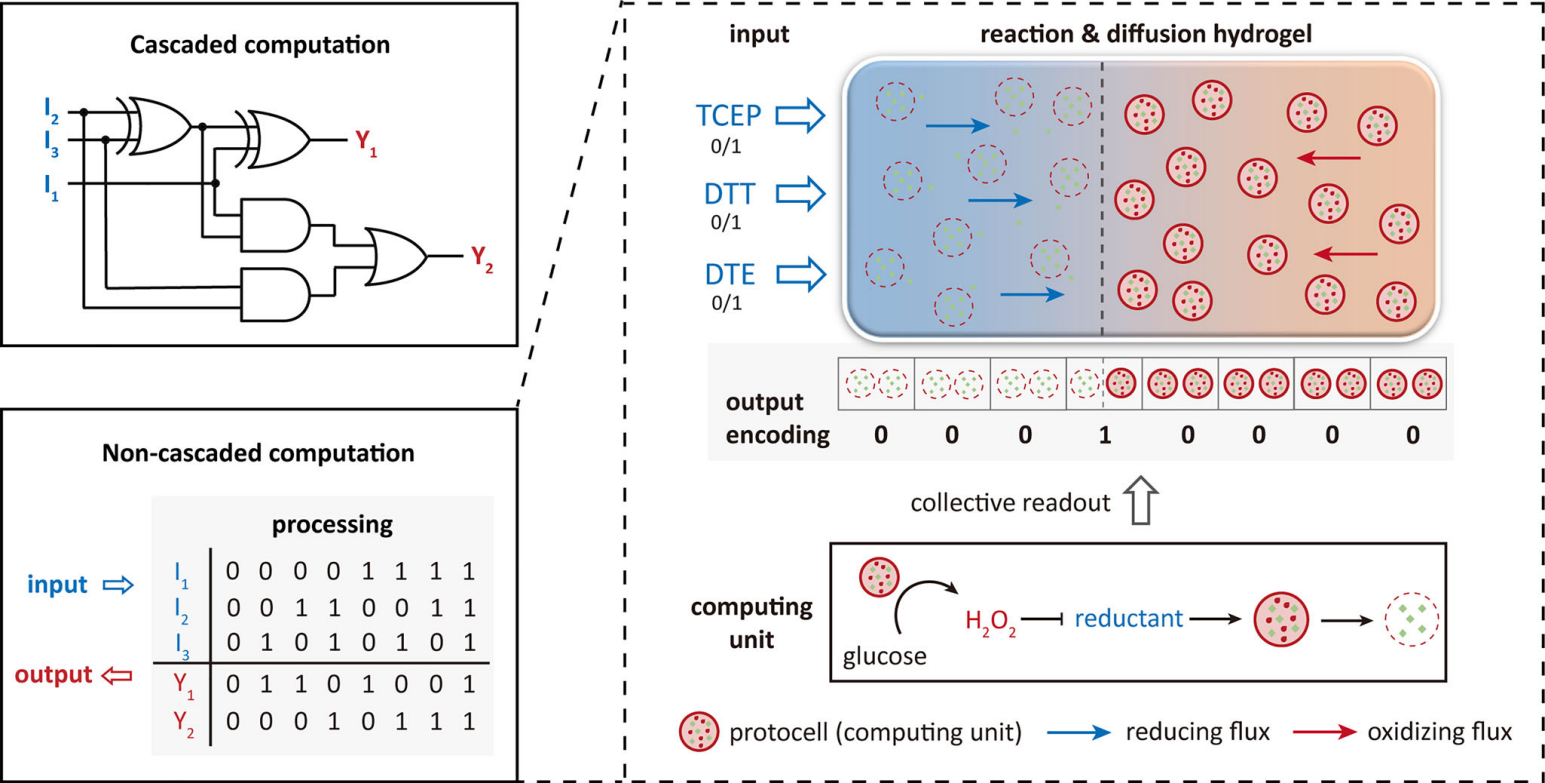
Conceptual design of the non‐cascaded spatial biocomputing architecture. The present non‐cascaded architecture uses a shared reaction–diffusion hydrogel field as the computing medium to directly map chemical inputs to spatially decoded outputs. A redox‐responsive population of GOx/RITC‐BSA co‐loaded proteinosomes was immobilized throughout the hydrogel as local computing units. Reductant inputs, including TCEP, DTT, and DTE, diffused from the left side, whereas glucose diffused from the right side and was converted by encapsulated GOx into H_2_O_2_ inside intact proteinosomes. The opposing reductive and oxidative fluxes regulated the extent of reductant‐induced proteinosome membrane disruption, thereby producing a spatial distribution of degraded and intact protocells. This population‐level spatial distribution was collectively read out as a reaction–diffusion boundary and further discretized by predefined output domains, in which the fluorescence boundary defined binary output states. Thus, computation was implemented as a direct mapping from chemical inputs to spatially encoded protocell states within a single reaction–diffusion field.

## 2 Results

### 2.1 Feedback‐Regulated Dissociation Kinetics in Redox‐Responsive Proteinosomes

To construct the proposed non‐cascaded computing architecture, we repurposed redox‐responsive proteinosomes as local protocellular response units that independently convert local chemical states into fluorescence outputs [28]. These proteinosomes consisted of semi‐permeable membranes assembled from protein–polymer nanoconjugates and contained RITC‐labeled BSA as a fluorescent reporter together with glucose oxidase (GOx) as an enzymatic component (Figure S1). The semi‐permeable membrane allows the diffusion of small molecules, including reductants, glucose and H_2_O_2_, while retaining encapsulated macromolecules under intact conditions. The disulfide bonds embedded within the proteinosome membrane undergo cleavage upon stimulation by reductants, such as tris(2‐carboxyethyl) phosphine (TCEP), dithiothreitol (DTT), and 1,4‐dithioerythritol (DTE), leading to the release of RITC‐labeled BSA encapsulated within the compartments upon structural disintegration (Figure 2A,B). These reductants act on the same redox‐responsive membrane motif, allowing the protocell response to be evaluated within a consistent chemical pathway. This process enables the readout of input signals (reductants) by monitoring the attenuation of intra‐compartmental fluorescence (protocell density: 7.2 × 10^5^ protocells/mL). Under identical reductant concentrations (final concentration: 0.25 mm), the total fluorescence intensity of the protocell population within the imaging field of view decreased to 20% within 9 min following DTT addition (Figure 2B,C). Here, the 20% fluorescence threshold was predefined as the kinetic readout, minimizing ambiguity caused by terminal noise, residual fluorescence, and minor baseline fluctuations near complete decay. In contrast, reaching the same population‐level fluorescence decrease required 20 min for DTE and 21 min for TCEP, respectively (Figure 2B,C), highlighting reductant‐specific dissociation kinetics. Consistent with this population‐level response, single‐protocell fluorescence analysis showed that individual protocells also underwent reductant‐induced fluorescence attenuation, with moderate cell‐to‐cell kinetic variation (Figure S2).

**FIGURE 2:**
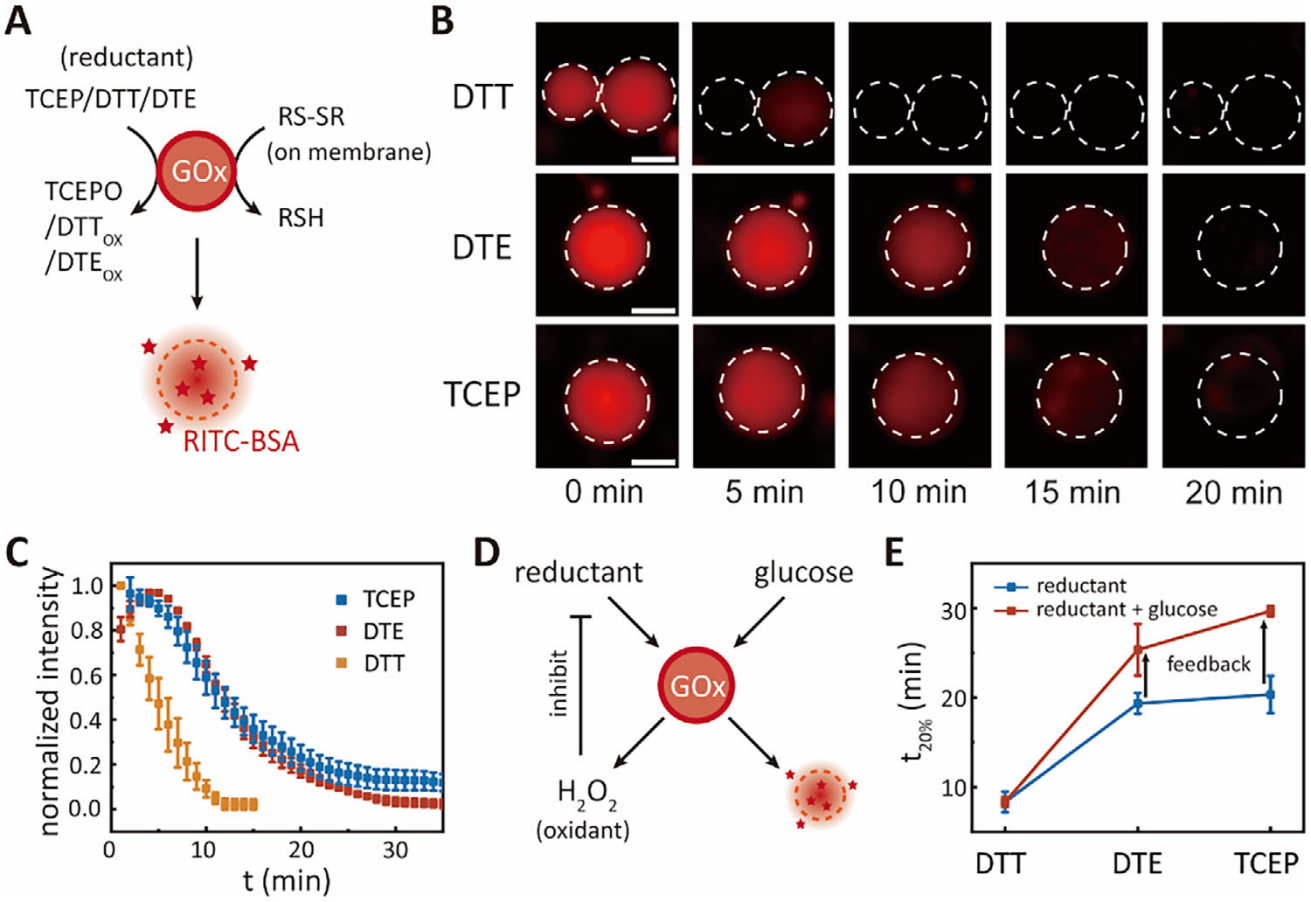
(A) Schematic of membrane disassembly and fluorescent cargo release in a GOx‐containing protocell model upon reductant exposure (e.g. TCEP; DTT; DTE). (B) Time‐dependent fluorescence microscopy images of GOx/RITC‐BSA‐loaded proteinosomes (7.2 × 10^5^ protocells/mL) upon exposure to DTT, DTE or TCEP (final concentrations: 0.25 mm). Scale bars: 50 µm. (C) Time‐dependent plots of the change of fluorescence intensity of (B). (D) Schematic of a negative feedback mechanism in which glucose‐driven H_2_O_2_ production by GOx inhibits reductant‐induced membrane rupture and cargo leakage. (E) Comparison of decay times (t_20%_) for GOx/RITC‐BSA‐loaded proteinosomes (7.2 × 10^5^ protocells/mL), where t_20%_ is the time for fluorescence intensity to drop to 20% of initial value. Data from time‐dependent fluorescence microscopy upon exposure to 0.25 mm DTT, DTE, or TCEP, alone or with 0.25 mm glucose.

Modulating the DTT/TCEP molar ratio (4:0, 3:1, 2:2, 1:3, and 0:4) resulted in a progressive increase in the time required for 80% fluorescence attenuation (t_20%_), from 9 min to 12, 16, 19 and 21 min, respectively (Figure S3). Similarly, varying the DTT/DTE ratio exhibited a consistent trend: as the proportion of DTT was decreased, the t_20%_ was systematically prolonged from 9 min to 12, 14, 18, and 20 min, respectively (Figure S3). Notably, mixtures of TCEP and DTE exhibited negligible variation in fluorescence decay kinetics (Figure S3), which could be due to the comparable intrinsic reduction kinetics of TCEP and DTE. Together, these results characterize the reductant‐dependent dissociation behavior of the proteinosome platform, providing the molecular response basis for the subsequent computing architecture.

To implement a negative feedback control loop, glucose oxidase (GOx) was encapsulated within the proteinosomes to trigger the enzymatic generation of H_2_O_2_ upon glucose addition, which serves as a scavenger for the input reductants (TCEP, DTT, and DTE) (Figure 2D and Figure S4). By exploiting the permeability of glucose across proteinosome membranes, the GOx‐encapsulated proteinosomes were incubated in equimolar solutions of reductant and glucose (final concentration: 0.25/0.25 mm). We observed that the regulatory efficiency of this feedback was intrinsically linked to the inherent reduction strength of the triggers. For the slower‐acting reductants, the presence of the glucose/GOx system significantly extended the t_20%_ from 19 to 25 min for DTE, and from 20 to 29 min for TCEP (Figure 2E and Figure S4). Interestingly, the dissociation profile triggered by DTT remained largely unchanged under the same feedback conditions (Figure 2E and Figure S4). This disparity suggests that at high reduction rates, the rapid cleavage of disulfide bonds outpaces the enzymatic scavenging of reductants. Consequently, the negative feedback loop acts as a kinetic filter that disproportionately delays slower dissociation processes, thereby magnifying the kinetic contrast between different chemical inputs.

### 2.2 Tunable Spatial Responses Based on a Randomly Distributed Protocell Network in Hydrogel Matrix

Protocells were then randomly immobilized within a rectangular hydrogel matrix to construct a 3D computing platform (Figure 3A), and each was encapsulated with RITC‐BSA and GOx, enabling a continuous spatial addressing profile along the longitudinal axis through fluorescence readout with a spatial binning interval of 30 µm at a protocell density of 3 × 10^5^ protocells/mL (Figure S5). In a logic computing device with dimensions of 8 × 4 × 2.5 mm, the reducing agent TCEP (500 mm, 2.5 µL) was introduced from the left side, while glucose (500 mm, 2.5 µL) was added to the right to maintain an oxidative environment. Proteinosomes in the hydrogel matrix were gradually disrupted along the diffusion direction of TCEP over time, resulting in leakage of internal fluorescence signals. In contrast, under the oxidative conditions on the right side, the proteinosome structures remained intact, and their internal fluorescence signals were preserved. Eventually, a stable reaction boundary was formed at approximately 4.2 mm from the left input site (Figure 3A,B and Figure S6). Replicate experiments performed under the same gradient conditions showed similar boundary displacement profiles, confirming the reproducibility of protocell dispersion and collective boundary formation within the hydrogel. Time‐lapse imaging over 12 h further showed that the boundary reached a quasi‐stable position after approximately 60 min, with only minor fluctuations thereafter (Figure S6). In control experiments without the glucose‐mediated feedback or under uniform conditions, all proteinosomes were completely disintegrated (Figure 3C,D and Figure S6). This comparison highlights that the imposed gradient enables the system to reach a stable non‐equilibrium steady state. Distinct from the uniform fate in homogeneous mixtures, the introduction of a spatial dimension enables the resolution and amplification of the underlying reaction kinetics. Furthermore, by varying the concentration of the reductant (TCEP: 31.25–500 mM) and the density of proteinosomes (0.75–6 × 10^5^ protocells/mL), tunability of the boundary position was demonstrated. Specifically, higher TCEP concentrations shifted the boundary toward the right in a natural logarithmic relationship. Lower proteinosome densities also resulted in rightward shifts (Figure S7), confirming that the spatial response boundary was continuously adjustable.

**FIGURE 3:**
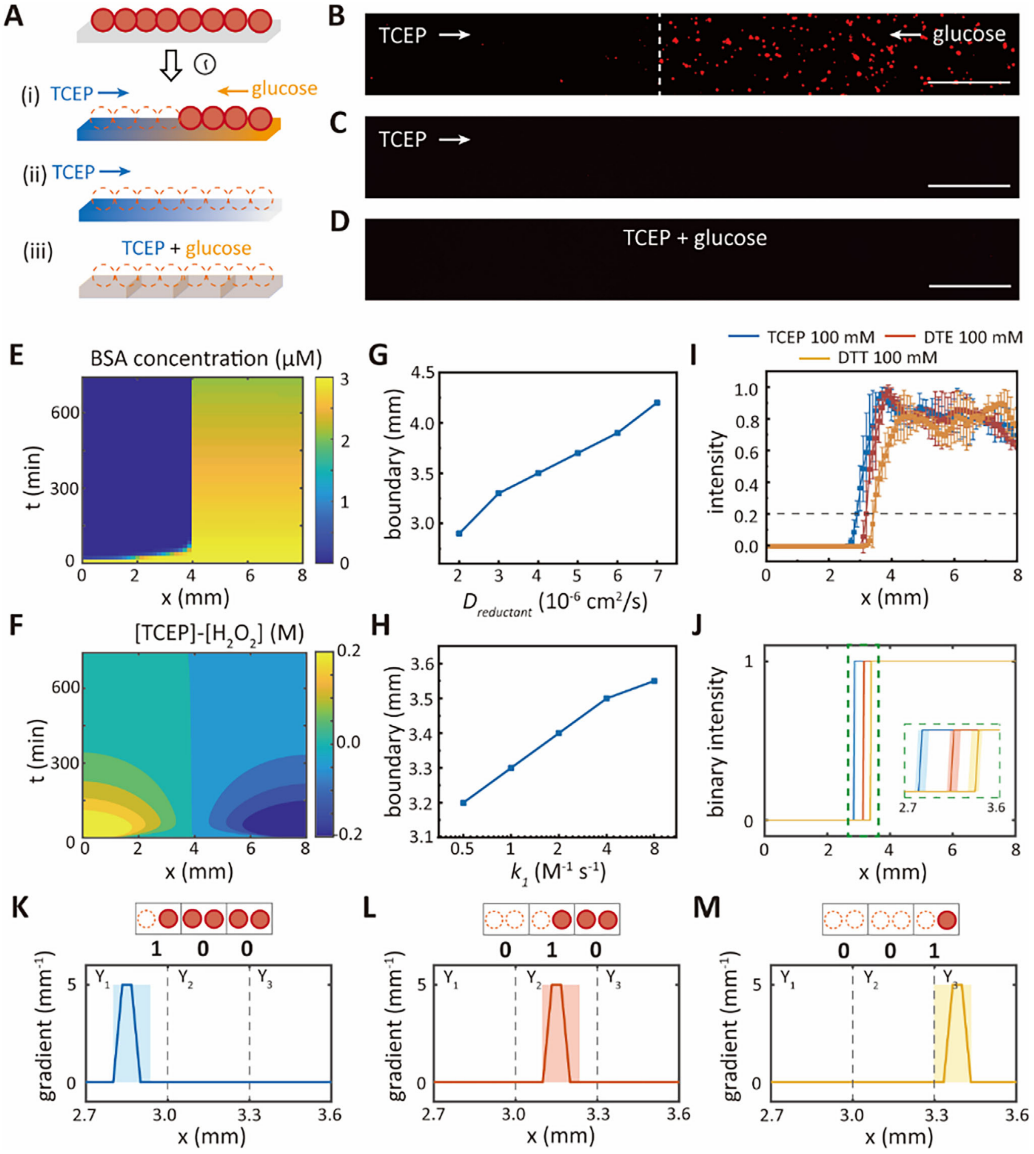
(A) Schematics of protocell responses in hydrogel‐immobilized, homogeneous GOx/RITC‐BSA‐loaded proteinosomes (3 × 10^5^ protocells/mL). Top: Initial state. (i) Opposing gradients of TCEP (left) and glucose (right) result in rupture on the left half and intact protocells on the right. (ii) TCEP gradient (left only) leads to complete rupture. (iii) Uniform addition of TCEP and glucose causes complete rupture. (B–D) Corresponding confocal fluorescence microscopy images recorded after exposure to: opposing gradients of TCEP (2.5 µL, 500 mm, left) and glucose (2.5 µL, 500 mm, right) for 150 min (B); TCEP (2.5 µL, 500 mm, left only) for 150 min (C); uniform TCEP (2.5 µL, 500 mm) and glucose (2.5 µL, 500 mm) for 10 min (D). Scale bars: 2 mm. (E, F) 2D plots of simulated spatiotemporal distribution of BSA concentration inside proteinosomes (E) and the concentration difference between TCEP and H_2_O_2_ ([TCEP] − [H_2_O_2_]) in hydrogel (F), under opposing gradients of TCEP (2.5 µL, 500 mm, left) and glucose (2.5 µL, 500 mm, right) in protocell‐containing hydrogel (3 × 10^5^ protocells/mL). (G) Plot of boundary position of BSA concentrations vs. artificially varied diffusion coefficients of reductant (*D_reductant_*), simulated in protocell‐containing hydrogel (3 × 10^5^ protocells/mL) under opposing gradients of reductant (1 µL, 500 mm, left) and glucose (1 µL, 500 mm, right). (H) Plot of boundary position vs. artificially varied reaction rate (*k_1_*) between reductant and disulfide bonds within proteinosomes, under opposing gradients of reductant (1 µL, 50 mm, left) and glucose (1 µL, 500 mm, right) in protocell‐containing hydrogel (3 × 10^5^ protocells/mL). (I) Plots of normalized fluorescence intensity in hydrogel‐immobilized, homogeneous GOx/RITC‐BSA‐loaded proteinosomes (3 × 10^5^ protocells/mL) recorded after 12 h, under opposing gradients of reductant (TCEP, DTE, or DTT; 1 µL, 100 mm, left) and glucose (1 µL, 500 mm, right). (J) Binary intensity profiles obtained by thresholding the normalized fluorescence profiles in (I) at 0.2. The reaction boundaries were located at 2.88 ± 0.06, 3.18 ± 0.06, and 3.42 ± 0.06 mm for TCEP, DTE, and DTT, respectively. The inset enlarges the 2.7–3.6 mm region, with shaded regions indicating the range of boundary positions from three independent experiments (*n* = 3). (K–M) Gradient profiles of the binary intensity in the 2.7–3.6 mm region for TCEP (K), DTE (L), and DTT (M), with shaded regions indicating the range of boundary positions from three independent experiments (*n* = 3). This region was divided into three output zones (Y_1_–Y_3_), and each zone was assigned “1” when a boundary‐associated gradient peak was detected within it and “0” otherwise.

A reaction–diffusion model was further applied to simulate the formation of this boundary (see Methods). The simulation results showed that the position of the boundary, which was correlated with BSA concentration, emerged at 70 min (approximately 4 mm) and remained stable thereafter (Figure 3E). By analyzing the spatiotemporal concentration profiles of TCEP and H_2_O_2_, it was found that the boundary was primarily determined by opposing diffusion gradients. TCEP diffused from the left, whereas glucose diffused from the right and was rapidly converted into H_2_O_2_ in intact GOx‐containing proteinosomes. The resulting H_2_O_2_ counter gradient impeded further TCEP diffusion and protected the proteinosomes on the right from degradation. Although GOx release during proteinosome disruption cannot be excluded, its contribution is expected to be limited under these conditions, as the glucose‐derived H_2_O_2_ field is established early, when membrane disruption remains spatially confined near the reductant‐input side (Figure 3F and Figure S8). The spatial position between intact and destroyed protocells was found to be a function of both the diffusion coefficient (*D_reductant_*) and the reduction rate constant (*k_1_*) of the input molecules. Specifically, the boundary shifted rightward as *D_reductant_* or *k_1_* increased (Figure 3G,H and Figure S9). By changing the concentrations of input reductant, we found that the boundary position was predominantly governed by the reaction rate at low input concentrations, whereas it became more sensitive to the diffusion rate at higher concentrations (Figure S10).

Given that DTT possesses a significantly higher reaction rate (*k_1_*) for disulfide cleavage compared to DTE and TCEP, and that both DTT and DTE exhibit faster diffusion rates (*D_reductant_*) than TCEP due to their lower molecular weights, we hypothesized that DTT would induce the most distal boundary displacement, followed by DTE and TCEP, within a certain range of input concentrations. Experimental results corroborated this hierarchy; by binarizing the longitudinal fluorescence intensity (threshold = 0.2), we localized the reaction boundaries at 2.88 ± 0.06, 3.18 ± 0.06, and 3.42 ± 0.06 mm for TCEP, DTE, and DTT, respectively, at a constant 100 mm input (Figure 3I,J and Figure S11). This trend remained robust across various concentrations (50 and 150 mm), confirming that the spatial reach of the input is a deterministic function of its molecular identity (Figure S12). Here, the coupling of diffusion with nonlinear feedback effectively amplifies the intrinsic physicochemical differences (both diffusivity and reactivity) of the inputs. By transforming these amplified variations into observable spatial outputs, the system achieves a higher resolution of chemical discrimination than would be possible in uniformly mixed conditions.

To interface these physical displacements with digital logic, we established a spatial encoding framework. As an illustrative demonstration, the active region (e.g. 2.7–3.6 mm of hydrogel) was partitioned into three discrete segments (Y_1_–Y_3_). By applying first‐derivative analysis to identify the binary fluorescence transition, the presence of a boundary was mapped to a digital “1” at a specific spatial address. Under this scheme, 100 mm TCEP, DTE, and DTT effectively “addressed” bits at Y_1_, Y_2_, and Y_3_, respectively (Figure 3K–M). These results establish chemically related reductants as a consistent input set whose shared disulfide‐cleavage‐mediated membrane‐disruption mechanism is converted into distinct spatial outputs through differences in effective reaction–diffusion kinetics. Building on this single‐input encoding, mixed‐reductant conditions further shifted the boundary relative to the corresponding single‐reductant inputs (Figure S13), showing that input composition can also be translated into a distinguishable spatial state. Thus, input identity, concentration and composition can be directly mapped to boundary positions and decoded by predefined spatial output regions within the same continuous reaction–diffusion field. In this framework, the feedback‐mediated inhibition of reductant activity provides the physicochemical basis for boundary stabilization, whereas the computation is implemented as non‐cascaded, parallel spatial readout rather than sequential reaction transfer.

### 2.3 Construction of a Half‐Adder Logic Computing Device Based on Spatial Encoding

A half‐adder logical computing device was then constructed, utilizing the reducing agents TCEP and DTT as binary input signals and the spatial positions of the responsive boundaries within the hydrogel matrix as outputs. This resulted in a two‐input (TCEP/DTT)—two‐output (Y_m_/Y_n_) half‐adder device. In this device, reducing‐agent inputs were introduced from the left side of an 8 × 4 × 2.5 mm proteinosome‐containing hydrogel chamber (3 × 10^5^ protocells/mL), while glucose was introduced from the right side to generate the opposing inhibitory field. Along the 8 mm x‐axis from the reductant‐input side, the hydrogel was divided into eight consecutive 1 mm‐wide spatial output regions, defined as Y_1_–Y_8_. Two of these spatial regions (Y_m_/Y_n_) were selected as the output regions for the half‐adder. When the response boundary was located within a selected output region, this output was defined as “1”; otherwise, it was defined as “0”. Four possible input combinations were defined for this half‐adder: the input “00” indicated that neither TCEP nor DTT was added; the input “01” signified the addition of only DTT; the input “10” represented the addition of only TCEP; and the input “11” denoted the simultaneous addition of both reducing agents. According to the half‐adder truth table, the two single‐input states, “01” and “10”, should activate only the sum‐output region (Y_n_), whereas the dual‐input state, “11”, should activate only the carry‐output region (Y_m_). Therefore, the expected outputs for “00”, “01”, “10”, and “11” are “0/0”, “0/1”, “0/1”, and “1/0”, respectively, when written as Y_m_/Y_n_, namely carry/sum (Figure 4A,B).

**FIGURE 4:**
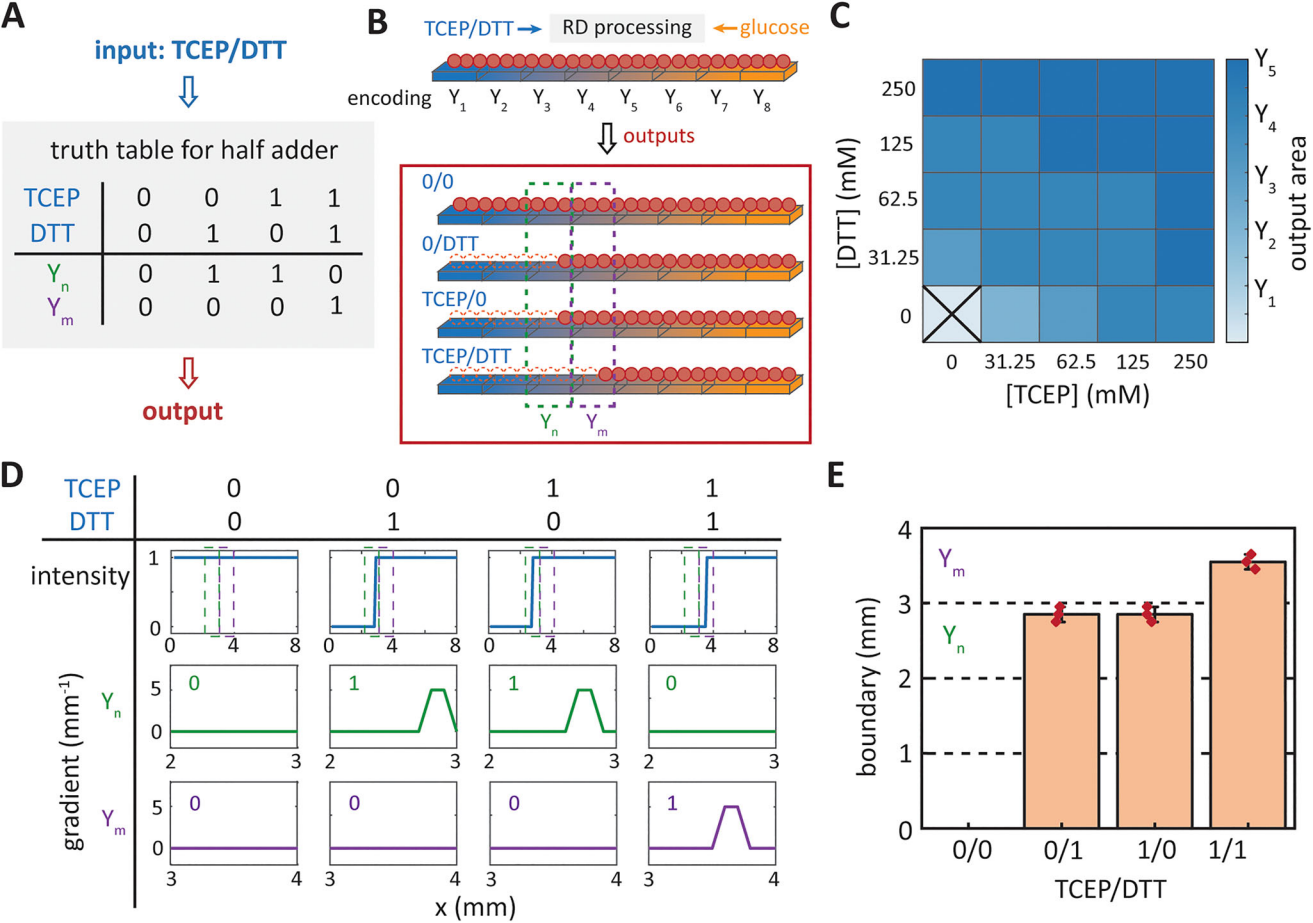
Design principle of the hydrogel‐based half‐adder operation. (A) Truth table for the half‐adder operation, where TCEP and DTT are used as two binary chemical inputs and Y_n_ and Y_m_ represent the two spatially decoded outputs. (B) Schematic illustration of the input‐to‐output mapping process. TCEP and/or DTT diffuse from the left side, while glucose diffuses from the right side, forming a shared reaction–diffusion (RD) field. The hydrogel is divided along the x direction into eight predefined spatial output regions, Y_1_–Y_8_, which serve as discrete readout zones for boundary‐position decoding. The four input combinations (TCEP/DTT: 0/0, 0/1, 1/0, 1/1; top to bottom) generate different boundary positions, which are assigned to the predefined output regions Y_n_ and Y_m_. The binary output (“1” or “0”) for each is determined by the presence of a signal peak within its assigned set of regions. (C) Discretized spatial output map obtained from the TCEP/DTT concentration matrix. The color scale indicates the output region, Y_1_–Y_5_, reached by the boundary under each concentration combination. Experiments were conducted in the proteinosome‐containing hydrogel (3 × 10^5^ protocells/mL) under varying TCEP/DTT gradients (0–250/0–250 mm, 2/2 µL, left) and a fixed glucose gradient (500 mm, 2 µL, right). (D) Composite plots depicting the binary response of the protocell‐containing hydrogel (3 × 10^5^ protocells/mL) to the four input combinations of a half‐adder. For each combination (TCEP/DTT in mM [logical value]: 0/0 [0/0], 0/31.25 [0/1], 62.5/0 [1/0], 62.5/31.25 [1/1]), a TCEP/DTT gradient was applied to the left (2/2 µL) and a fixed glucose gradient (500 mm, 2 µL) to the right. Top: binary intensity plots. Middle: gradient of binary intensity in region Y_n_ (2–3 mm, Y_3_). Bottom: gradient of binary intensity in region Y_m_ (3–4 mm, Y_4_). (E) Bar chart of boundary positions extracted from the protocell responses under the same input conditions as in (D). The TCEP/DTT concentrations in mm were 0/0, 0/31.25, 62.5/0, and 62.5/31.25, corresponding to the logical inputs [0/0], [0/1], [1/0], and [1/1], respectively. The corresponding boundary positions were 0 ± 0, 2.85 ± 0.10, 2.85 ± 0.10, and 3.55 ± 0.10 mm (*n* = 3).

To convert chemically induced boundary displacement into discrete half‐adder outputs, we first mapped the relationship between TCEP/DTT concentrations, boundary positions, and predefined spatial output regions using a TCEP (0–250 mm)/DTT (0–250 mm) concentration matrix experiment (Figure 4C). This mapping was used to identify a concentration window in which single‐input and dual‐input states could be assigned to distinguishable output regions within Y_1_–Y_8_. Under 31.25 mm TCEP, as the DTT concentration increased from 0 to 62.5 mm, the responsive interval transitioned from Y_2_ (1.9 mm) to Y_4_ (3.7 mm). When the DTT concentration was further increased to 250 mm, the responsive region shifted to Y_5_ (4.5 mm), indicating the different spatial responses between a single TCEP input and a combined TCEP‐DTT input. Similarly, under a 62.5 mm TCEP condition, as the DTT concentration increased from 0 to 250 mm, the responsive region gradually migrated from Y_3_ (2.7 mm) to Y_5_ (4.7 mm). At high TCEP conditions (125/250 mm), the effect of DTT concentration changes on the responsive region became less pronounced, resulting in responsive regions that were confined to only two distinct spatial segments (Figure S14). Additionally, when the DTT concentration was fixed at 0/31.25 mm, adjusting the TCEP concentration within the range of 0–250 mm led to dynamic changes in three different responsive intervals. In contrast, under another DTT concentration (62.5/125 mm), only two responsive intervals were observed, and at higher DTT concentrations (250 mm), almost no responsive displacement occurred (Figure S14). These experiments showed that the responsive boundary could be progressively displaced by changing the concentration ratio of TCEP and DTT, allowing different chemical input combinations to be converted into discrete spatial outputs.

According to the encoding characteristics of the half‐adder, the output regions triggered by a single input (TCEP or DTT) and a dual input (TCEP + DTT) should be distinguishable. Therefore, 62.5 mm TCEP was selected as the first “1” input concentration. Under this condition, the addition of different DTT concentrations (31.25–250 mm) could elicit significant changes in the output region. This was because each single input activated the Y_3_ region separately, while their synergistic action caused the responsive boundary to migrate to the Y_4_ region. Based on this characteristic, TCEP (0/62.5 mm) and DTT (0/31.25 mm) were ultimately chosen as the input signals, and Y_4_ and Y_3_ were selected as the output regions, successfully achieving the logical operation function of the half‐adder (Figure 4D and Figure S15). Specifically, the input “00” (TCEP/DTT: 0/0 mm) corresponded to an output of “00” (Y_4_/Y_3_: 0/0); the input “01” (TCEP/DTT: 0/31.25 mm) corresponded to an output of “01” (Y_4_/Y_3_: 0/1); the input “10” (TCEP/DTT: 62.5/0 mm) also corresponded to an output of “01” (Y_4_/Y_3_: 0/1); and the input “11” (TCEP/DTT: 62.5/31.25 mm) yielded an output of “10” (Y_4_/Y_3_: 1/0) (Figure 4E). These results demonstrate that the TCEP/DTT input combinations could be translated into discrete spatial boundary outputs, thereby producing the input–output relationship of a half‐adder within a single proteinosome–hydrogel reaction–diffusion field.

### 2.4 Construction of a Full‐Adder Logic Computing Device Based on Spatial Encoding

Moreover, a three‐input, two‐output full adder was implemented within a hydrogel‐based computational device (protocell: 3 × 10^5^ protocells/mL, 20 × 5 × 2 mm). Three reducing agents (TCEP, DTT, and DTE) were used as inputs, and the positions of diffusion‐induced boundaries were utilized as logical outputs (Y_n_/Y_m_). The hydrogel device was divided longitudinally into 20 consecutive output segments with a width of 1 mm each (Y_1_–Y_20_). The left segment group (Y_1_–Y_10_, 0–10 mm) was assigned to encode the sum bit (Y_n_), whereas the right segment group (Y_11_–Y_20_, 10–20 mm) was designated to encode the carry bit (Y_m_). A “1” was recorded when a boundary was detected in the corresponding region; otherwise, the output was defined as “0”. Based on the truth table of a full adder, four possible output combinations (“00”, “01”, “10”, and “11”) are generated from all permutations of the three binary inputs: inputs “001”, “010”, and “100” yield output “10” (Y_n_/Y_m_, 1/0); inputs “011”, “101”, and “110” yield output “01” (Y_n_/Y_m_, 0/1); input “111” yields output “11” (Y_n_/Y_m_, 1/1); input “000” yields output “00” (Y_n_/Y_m_, 0/0) (Figure 5A,B).

**FIGURE 5:**
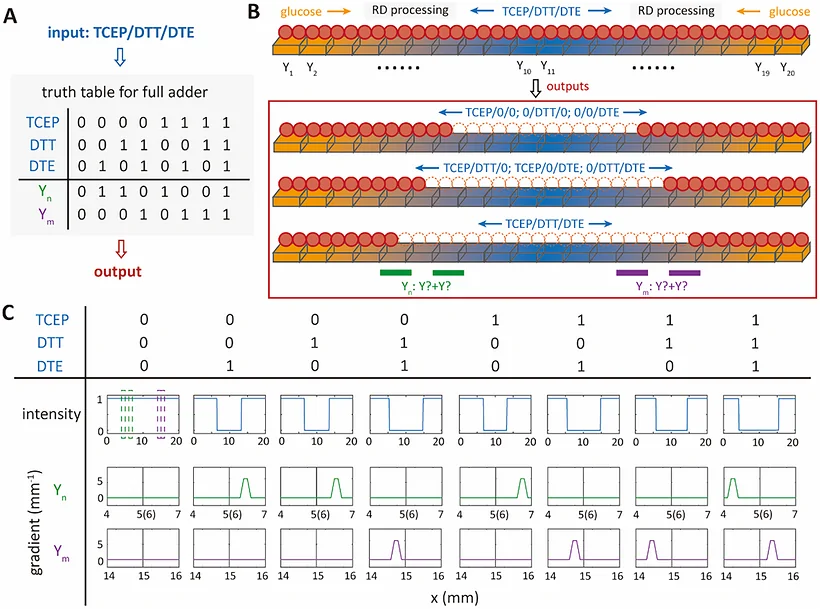
Design principle of the hydrogel‐based full‐adder operation. (A) Truth table for the full‐adder, where TCEP, DTT, and DTE serve as three binary chemical inputs, and Y_n_ and Y_m_ represent the spatially decoded sum and carry‐out outputs, respectively. (B) Schematic illustration of the parallel input‐to‐output mapping process. The proteinosome‐containing hydrogel is divided along the x direction into twenty predefined spatial regions, Y_1_–Y_20_, which serve as discrete readout zones for boundary‐position decoding. TCEP, DTT, and/or DTE inputs are introduced into the central region of the hydrogel, while glucose is supplied from both the left and right sides. This configuration establishes two reaction–diffusion (RD) processes that operate in parallel on the two sides of the central input region. Under the eight input combinations, different degrees of central proteinosome disruption generate distinct fluorescence patterns, which are assigned to the predefined output regions (Y_n_/Y_m_). The binary output (“1” or “0”) for each bit is determined by the presence of a signal peak within its assigned set of regions. (C) Composite analysis of the hydrogel‐based full‐adder (protocell density: 3 × 10^5^ protocells/mL) under all eight input conditions (Inputs: TCEP/DTT/DTE; “1” = 50/25/50 mm, 2 µL, central; “0” = absent). Each column shows, from top to bottom: the binary intensity map; the gradient in the sum‐defining regions (Y_5_ and Y_7_, where a signal in either region yields Y_n_ = 1); and the gradient in the carry‐out‐defining regions (Y_15_ and Y_16_, where a signal in either region yields Y_m_ = 1). Fixed glucose gradients (500 mm, 2 µL) were applied on both sides.

To enable independent spatial encoding of both outputs, TCEP, DTT, and DTE were introduced at the center of the hydrogel device, while glucose solution (500 mm, 2 µL) was fixedly loaded at both ends. The interaction between the diffusing input species and the glucose solutions led to the formation of two distinct chemical boundaries, which served as distributed logic readouts. Upon introduction of 50 mm TCEP (input “100”), a boundary was observed at position Y_7_ on the left side and at Y_14_ on the right side. Consequently, Y_7_ was assigned as the effective interval for Y_n_, whereas Y_14_ was excluded from the effective range for Y_m_. To ensure consistent output behavior for inputs “010” and “100”, 25 mm DTT and 50 mm DTE were selected as alternative inputs. These conditions also produced boundaries at Y_7_ (for Y_n_) and Y_14_ (not for Y_m_), thereby generating the same “10” output across all three cases (Figure 5C and Figure S16).

When TCEP (50 mm) and DTT (25 mm) were applied together (input “110”), the left boundary shifted to Y_6_ and the right boundary moved to Y_15_. Under these conditions, Y_6_ was not included in the Y_n_ readout window, while Y_15_ was identified as part of the Y_m_ response interval, resulting in an output of “01”. Consistent boundary positions (Y_6_ and Y_15_) were observed for inputs “011” and “101”, confirming the reliability of the “01” output across these three input combinations (Figure 5C and Figure S17). For input “111”, where all three reagents were introduced simultaneously, the left boundary shifted further forward to Y_5_ and the right boundary extended to Y_16_. These positions were recognized as the effective intervals for Y_n_ and Y_m_, respectively, yielding output “11”. In the absence of any input (i.e., input “000”), no boundary was detected on either side, resulting in output “00” (Figure 5C and Figures S18 and S19).

## 3 Conclusion

In summary, we have developed a programmable, non‐cascaded biocomputing platform based on an environment‐responsive proteinosome–hydrogel composite. In contrast to conventional DNA strand‐displacement circuits and enzyme‐cascade logic systems, where input–output relationships are typically encoded through molecular recognition, orthogonal signal design, and sequential reaction modules [60, 61], the present system utilizes a continuous reaction–diffusion field to directly translate chemical inputs into spatially resolved material responses [48, 62]. By employing reducing agents (TCEP, DTT, DTE) as input signals and utilizing glucose to establish a biochemical feedback network, we achieved precise control over stable spatiotemporal response boundaries by modulating reductant concentrations and protocell densities within opposing diffusion gradients. Crucially, the synergy between the feedback network and gradient response accentuates the subtle disparities in the reaction‐diffusion kinetics of diverse input molecules, translating them into distinct spatial patterns. This mechanism facilitates a multi‐bit spatial encoding system characterized by non‐overlapping output channels across the spatial domain, where the logical output is determined by the heterogeneous distribution of protocells within these channels. Through the reconfiguration of logical mapping, we successfully demonstrated the parallel execution of complex operations, including half‐adders and full‐adders.

These features also define the current limitations of the system. Its response speed is constrained by millimetre‐scale diffusion and boundary stabilization in the hydrogel, and the membrane‐disruption‐based readout makes the operation non‐resettable. Thus, the present system is better regarded as a proof‐of‐concept protocellular reaction–diffusion platform that demonstrates how the intrinsic physicochemical differences of chemical inputs can be converted into logic outputs through a spatially encoded, non‐cascaded architecture. Future work may improve its performance through device miniaturization, microfluidic gradient control, and reversible or non‐destructive readout designs [63, 64]. With further expansion of spatiotemporal input dimensions and spatial readout regions, this framework may provide opportunities for more complex spatial classification, multi‐input chemical discrimination, and pattern‐recognition tasks [65].

Overall, this work underscores the potential of redox‐responsive protocells for chemical computation and information processing, offering insights into designing advanced biomimetic systems with programmable and tunable functionalities. This integrated framework merges biochemical dynamics, materials science, and computational theory, presenting a new paradigm for advancing biocomputing toward enhanced adaptability and programmability. By bridging synthetic biology and computational sciences, our approach lays the groundwork for next‐generation bio‐inspired information processing systems.

## Author Contributions

L.T. conceptualized and supervised the project; S.W. performed all experiments and simulations; all the authors undertook the data analysis; S.W. and L.T. wrote the manuscript. All authors read and approved the final manuscript.

## Conflicts of Interest

The authors declare no conflicts of interest.

## Supporting information




**Supporting File**: advs77019‐sup‐0001‐SuppMat.docx.

## Data Availability

The data that support the findings of this study are available from the corresponding author upon reasonable request.
