# Supplementary material for: Spatially Encoded Protocell Network for Non‐Cascaded Parallel Biocomputation
Source: Adv Sci (Weinh). 2026 Aug 18;13(62):e77019. doi: 10.1002/advs.77019 (PMC13482934; doi:10.1002/advs.77019)
Supplement: Supplementary file 1 [file ADVS-13-e77019-s001.docx]

**Supporting information**

**Spatially Encoded Protocell Network for Non-Cascaded Parallel Biocomputation**

**Shuqi Wu^1^, Liangfei Tian^1,2,3*^**

*1. Zhejiang Key Laboratory of Intelligent Sensing Technology and Advanced Medical Instrument, Key Laboratory of Biomedical Engineering of Ministry of Education, College of Biomedical Engineering & Instrument Science, Zhejiang University, Hangzhou, 310027, China*

*2. Innovation Center for Smart Medical Technologies & Devices, Binjiang Institute of Zhejiang University, Hangzhou, 310053, China*

*3. Department of Ambulatory Surgery, Women's Hospital,* *School of Medicine, Zhejiang University, Hangzhou, 310027, China*

*Correspondence to:* *[liangfei.tian@zju.edu.cn](mailto:liangfei.tian@zju.edu.cn)*

**1. Materials and methods**

**Materials.** Agarose (congealing temperature 26-30°C), acetonitrile, azobisisobutyronitrile (AIBN, ≥99%), bovine serum albumin (BSA), glucose oxidase (GOx, 158.2 U mg^−1^), 4, 7, 10, 13, 16, 19, 22, 25, 32, 35, 38, 41, 44, 47, 50, 53-hexadecaoxa-28, 29-dithiahexapentacontanedioic acid di-N-succinimidyl ester (NHS-PEG16-DS, ≥94%), 1,6-hexanediamine (HMDA, ≥98%), N-ethyl-N′-(3-dimethylaminopropyl) carbodiimide hydrochloride (EDAC, ≥98%), N-hydroxysuccinimide 2-(dodecylthiocarbonothioylthio)isobutyrate (RAFT, 98%), N-isopropyl acrylamide (NIPAAm, ≥99%), rhodamine isothiocyanate (RITC, ≥99%), and tris(2-carboxyethyl)phosphine hydrochloride (TCEP, ≥98%) were purchased from Sigma-Aldrich. Dithioerythritol (DTE, ≥99%) and Dithiothreitol (DTT, ≥99%) were purchased from Aladdin. Dialysis bags with MWCO 12-14 kDa were purchased from Millipore.

**Fluorescence and confocal microscopy experiments.** Fluorescence microscopy images were acquired using a Leica DMI8 inverted microscope. Confocal fluorescence microscopy was conducted using an Olympus FV3000 confocal laser scanning microscope (CLSM). Fluorescence images were acquired with an excitation wavelength of 550 nm and emission wavelength of 580-620 nm, corresponding to the RITC (Rhodamine isothiocyanate) fluorescence dye. For large-area imaging, the MATL module was combined with an automatic image stitching module to capture multiple tiled regions, covering the entire sample area. These individual image tiles were then automatically stitched together to produce a wide-field composite image.

**Preparation of** **fluorescently labelled enzymes.** Fluorescently labelled enzymes were prepared following a previously reported procedure^[1]^. Protein solutions (BSA, 4 mg/mL) were prepared by dissolving commercially available BSA in 10 mL of 0.1 M carbonate buffer (pH 8.5). An aliquot of freshly prepared RITC (10 mg/mL, 10 μL) in anhydrous DMSO was added dropwise to the protein solution. The reaction mixture was kept in the dark at 4°C overnight, then dialyzed against Milli-Q water and freeze-dried.

**Synthesis of PNIPAAm.** PNIPAAm was prepared following a previously reported procedure^[1]^. To synthesize PNIPAAm, RAFT (18.9 mg, 50 μmol), AIBN (1.7 mg, 10 μmol), NIPAAm (847 mg, 7.5 mmol), and acetonitrile (8 mL) were added to a round-bottom flask. The solution was degassed by four freeze-pump-thaw cycles and polymerized at 60°C for 8 hours. The product was purified by precipitation in diethyl ether/hexane (2:1 ratio). The molecular weight of PNIPAAm was determined by ¹H NMR spectroscopy, yielding an average molecular weight of 7100 g/mol^[1]^.

**Synthesis of BSA-PNIPAAm nanoconjugate.** BSA-PNIPAAm nanoconjugate was prepared following a previously reported procedure^[1]^. BSA was cationized using 1,6-hexanediamine (HMDA). An aqueous HMDA solution (100 mg/mL, 5 mL) was added dropwise to an aqueous solution of BSA (20 mg/mL, 5 mL), and the coupling reaction was initiated with EDAC (50 mg). The pH was maintained at 6.5 by adding aliquots of 0.1 M HCl, and an additional 50 mg of EDAC was added after 5 h. After stirring overnight and dialysis (MWCO 12-14 kDa) against Milli-Q water, the solution was freeze-dried. An aqueous solution of PNIPAAm (2 mg/mL, 5 mL) was then added dropwise to the cationized BSA solution (2 mg/mL, 5 mL) prepared in 0.1 M Na_2_CO_3_ buffer at pH 8.5. The mixture was stirred at 4°C for 12 hours. The product was purified by ultrafiltration (MWCO 50 kDa), and analyzed by MALDI-TOF MS, confirming the attachment of approximately 3 polymer chains per BSA molecule^[1]^.

**Proteinosome preparation.** Proteinosomes were prepared following a previously reported procedure^[1]^. An aqueous solution of BSA-PNIPAAm nanoconjugates (8 mg/mL, 30 μL) were mixed with a buffered solution (Na_2_CO_3_, 0.1 M, pH 8.5) containing cargo proteins (RITC-BSA/GOx, 10 mg/mL each, 25 μL). An aqueous solution of NHS-PEG16-DS cross-linker (40 mg/mL, 5 μL) was added to the mixture, followed by the addition of 1 mL of 2-ethyl-1-hexanol. The solution was vortexed for 30 seconds to form a water-in-oil emulsion. After 12 hours of sedimentation, the oil phase was discarded, and the proteinosomes were washed with 75% ethanol twice and Milli-Q water once through centrifugation.

**Determination of proteinosome concentrations.** Proteinosome concentration was determined using a hemocytometer. A 10 μL sample of proteinosome solution was loaded into the hemocytometer, and the number of proteinosomes (n) in the central large square was counted under a microscope. This process was repeated three times, and the concentration was calculated as n × 10^4^ particles/mL.

**Fluorescence intensity changes in protocells under homogenous redox conditions.** The reduction kinetics induced by different combinations of reducing agents were systematically examined. Specifically, a solution of GOx/RITC-BSA-loaded proteinosomes (9 × 10^5^ protocells/mL, 45 μL) was mixed with varying volume ratios of TCEP and DTT mixtures (TCEP/DTT: 2.5/2.5 mM; volumes: 0/5, 1.25/3.75, 2.5/2.5, 3.75/1.25, 5/0 μL; pH 8.5) across five separate experimental sets. The resulting mixture was loaded into a custom-made capillary slide for fluorescence microscopy imaging using a 20× objective. After a 4 min settling period for the protocells, time-lapse fluorescence images were acquired at 60 s intervals. To prevent sample evaporation, the slide was sealed with vacuum grease. The same procedure was repeated to evaluate the reduction kinetics in the presence of TCEP and DTE mixtures (TCEP/DTE: 2.5/2.5 mM; volumes identical to those above; pH 8.5), and subsequently for DTT and DTE mixtures (DTT/DTE: 2.5/2.5 mM; volumes identical; pH 8.5), with all other experimental parameters kept consistent. Additionally, to validate the negative feedback kinetics, parallel experiments were conducted using TCEP, DTT, or DTE mixed with glucose (each at 5 mM; 2.5 μL added per agent) under otherwise identical imaging and sample-handling conditions. Each condition was repeated three times. For each individual repeat, TCEP, DTT, or DTE stock solutions were freshly prepared immediately before use, and the pH of each reductant stock solution was adjusted to 8.5 prior to addition. Replicate measurements for the same condition were performed on the same day to minimize variations associated with protocell preparation, hydrogel embedding, and possible changes in reductant activity during storage or exposure to air.

For population-level fluorescence analysis, background subtraction was first performed for each frame of the time-lapse fluorescence images. All detectable protocell regions within the field of view were then extracted, and the total fluorescence intensity from these regions was calculated at each time point. The total fluorescence intensity was normalized to the initial value to obtain the fluorescence attenuation curves of the protocell population under homogeneous redox conditions. For single-protocell fluorescence analysis, approximately 20 individual protocells were manually selected as regions of interest (ROIs) from representative time-lapse images using ImageJ. The fluorescence intensity within each ROI was measured frame by frame and normalized to the initial fluorescence intensity of the corresponding protocell. The time required for the fluorescence intensity to decrease to 20% of its initial value was used as a quantitative descriptor of single-protocell response kinetics.

**Preparation and large-area confocal imaging of proteinosome-immobilized hydrogel.** To prepare proteinosome-immobilized hydrogel, a GOx/RITC-BSA loaded proteinosomes solution (5 × 10^5^ protocells/mL, 48 μL) was mixed with agarose solution (0.2 wt%, 32 μL). A total of 80 μL of protocell-containing hydrogel (3 × 10^5^ protocells/mL) was then loaded into a rectangular chamber (L/W/H: 8/4/2.5 mm), which was cut from an acrylic plate and adhered to a coverslip at the bottom. The entire setup was placed in a confocal dish filled with water to prevent dehydration of the hydrogel. Large-area fluorescence imaging was performed using an Olympus confocal microscope equipped with a 10× objective. The entire 8 × 4 mm hydrogel region was acquired as a 7 × 4 tile scan. Adjacent image tiles contained approximately 10% overlap, and the scan was performed using a serpentine pattern.

The exported TIFF tiles were stitched in MATLAB. The seven tiles in each row were assembled according to their respective scan directions, with the overlapping regions between adjacent tiles removed during stitching. The four row-wise stitched images were then combined from top to bottom, with the vertical overlaps between adjacent rows also removed, to generate the full mosaic image. The stitched image was subsequently processed in ImageJ for figure presentation using Gaussian filtering (sigma = 2 pixels), background subtraction with a rolling-ball radius of 50 pixels, automatic contrast adjustment, and cropping to the hydrogel region. For spatial fluorescence profiling, the hydrogel image was divided into consecutive bins along the longitudinal axis, with the bin width selected according to the specific analysis. The fluorescence signal within each bin was then quantified to generate the spatial fluorescence profile.

**Time-lapse imaging and image analysis for boundary formation in proteinosome-immobilized hydrogel.** Proteinosome-immobilized hydrogels were prepared as described above in rectangular acrylic chambers (L/W/H: 8/4/2.5 mm) at a fixed protocell density of 3 × 10^5^ protocells mL^-1^. For the feedback experiment, TCEP solution (500 mM, 2.5 μL, pH 8.5) and glucose solution (500 mM, 2.5 μL) were added to the left and right sides of the hydrogel, respectively. Time-lapse fluorescence imaging was performed using a 10× objective on a confocal microscope. At each time point, the entire 8 × 4 mm hydrogel region was imaged as a 7 × 4 stitched mosaic, with each field acquired at 512 × 512 pixels. The acquisition and stitching time for one complete stitched image was approximately 1.5 min. Images were collected every 6 min for 12 h, and the experiment was repeated three times. Independent repeats were performed on different days using newly prepared reductant stock solutions and freshly prepared proteinosome-containing hydrogels. For the no-feedback control, TCEP solution (500 mM, 2.5 μL, pH 8.5) was added to the left side of the hydrogel, while Milli-Q water (2.5 μL) was added to the right side. Images were acquired every 3 min for 3 h using the same imaging settings.

Image preprocessing was carried out in ImageJ. Fluorescence images were filtered using a 2D Gaussian filter (sigma = 2 pixels), followed by background subtraction. Protocell regions were segmented using the global Otsu thresholding algorithm, and the resulting binary images were used as masks for subsequent fluorescence analysis in MATLAB. The fluorescence images and corresponding masks were imported into MATLAB, where protocell-associated fluorescence was extracted by mask-based multiplication. The hydrogel region was divided into 80 bins along the x-axis, each corresponding to a 0.1 × 4 mm region, and the total fluorescence intensity within each bin was summed to obtain a spatial fluorescence profile.

For boundary analysis, the spatial fluorescence profiles were smoothed and normalized to the initial fluorescence intensity. The normalized profiles were binarized using a threshold of 0.2, defining regions with fluorescence intensity below 20% as disrupted protocell regions. The boundary position was determined from the peak of the differentiated binary profile calculated using the MATLAB gradient function. Time-dependent two-dimensional fluorescence maps were visualized using the MATLAB pcolor function.

**Matrix screening of protocell density and reductant concentrations in hydrogel-immobilized protocell systems.** Proteinosome-immobilized hydrogels were prepared as described above in rectangular acrylic chambers (L/W/H: 8/4/2.5 mm) at a fixed protocell density of 3 × 10^5^ protocells mL^-1^. To systematically evaluate the effects of protocell density and TCEP concentration, a matrix experiment was performed. The protocell density in the hydrogel was varied as 0.75, 1.5, 2.25, 3, 4.5 and 6 × 10^5^ protocells/mL, while the left-side TCEP solution was tested at concentrations of 31.25, 62.5, 125, 250 and 500 mM (1 μL). The right side consistently received glucose solution (500 mM, 1 μL). This resulted in a total of 30 experimental conditions. Each condition was repeated three times. In a separate matrix experiment designed to examine the interplay between TCEP and DTT gradients, the protocell density within the hydrogel was maintained at 3 × 10^5^ protocells/mL. The right side of the hydrogel received a fixed concentration of glucose solution (500 mM, 1 μL). On the left side, the concentrations of TCEP and DTT solutions used were as follows: TCEP (0/31.25/62.5/125/250 mM, 1 μL) and DTT (0/31.25/62.5/125/250 mM, 1 μL), resulting in a total of 25 experimental conditions. Each condition was repeated three times. For each individual repeat, TCEP or DTT stock solutions were freshly prepared before use, and the pH of each reductant stock solution was adjusted to 8.5 prior to addition. Replicate measurements for the same condition were performed on the same day to minimize variations associated with protocell preparation, hydrogel embedding, and possible changes in reductant activity during storage or exposure to air.

After 12 h of incubation, wide-field fluorescence images of the hydrogel were acquired using confocal microscopy for each condition. The acquisition and stitching time for one complete stitched image was approximately 1.5 min. Image preprocessing and boundary extraction were performed as described above. Briefly, the hydrogel region was divided into 40 bins along the x-axis, each corresponding to a 0.2 × 4 mm region. The protocell-associated fluorescence intensity within each bin was summed to obtain a spatial fluorescence profile, which was then smoothed, normalized and binarized using a threshold of 0.2. Boundary positions were determined from the peak of the differentiated binary profile calculated using the MATLAB gradient function.

**Boundary formation induced by TCEP, DTT and DTE at identical concentrations.** Proteinosome-immobilized hydrogels were prepared as described above in rectangular acrylic chambers (L/W/H: 8/4/2.5 mm) at a fixed protocell density of 3 × 10^5^ protocells mL^-1^. To compare the reaction-diffusion profiles of TCEP, DTT, and DTE, the assay was conducted with a fixed protocell density (3 × 10^5^ cells/mL) and opposing gradients: 500 mM glucose (1 µL) on the right and 1 µL of each reductant at 50, 100, or 150 mM on the left. In a separate combined-reductant experiment, TCEP, DTT, and DTE were simultaneously introduced on the left side of the hydrogel, with each reductant added at 50 mM in a volume of 1 µL, while the right side received the same glucose input of 500 mM glucose (1 µL). Each condition was repeated three times. For each individual repeat, TCEP, DTT, or DTE stock solutions were freshly prepared before use, and the pH of each reductant stock solution was adjusted to 8.5 prior to addition. Replicate measurements for the same condition were performed on the same day. After 12 h of incubation, wide-field fluorescence images were acquired using confocal microscopy. The acquisition and stitching time for one complete stitched image was approximately 1.5 min. Image preprocessing and boundary extraction were performed as described above. Briefly, the hydrogel region was divided into 80 bins along the x-axis, each corresponding to a 0.1 × 4 mm region. The protocell-associated fluorescence intensity within each bin was summed to obtain a spatial fluorescence profile, which was then smoothed, normalized and binarized using a threshold of 0.2. Boundary positions were determined from the peak of the differentiated binary profile calculated using the MATLAB gradient function.

**Half-adder logic computing in protocell collective responses.** Proteinosome-immobilized hydrogels were prepared as described above in rectangular acrylic chambers (L/W/H: 8/4/2.5 mm) at a fixed protocell density of 3 × 10^5^ protocells mL^-1^. A fixed glucose solution (500 mM, 1 μL) was added at the right side of the hydrogel. A combined gradient of TCEP and DTT solutions was introduced at the left side of the hydrogel, according to four distinct conditions. The reducing agent inputs consisted of TCEP and DTT at the following concentration combinations (TCEP/DTT: 0/0; 0/31.25; 62.5/0; 62.5/31.25), with a total volume of 2 μL added for each agent (resulting in a combined volume of 4 μL for double-agent conditions). Each condition was repeated three times. For each individual repeat, TCEP or DTT stock solutions were freshly prepared before use, and the pH of each reductant stock solution was adjusted to 8.5 prior to addition. Replicate measurements for the same condition were performed on the same day. After 12 hours of incubation, wide-field images of the hydrogel were captured for each condition using confocal microscopy. The acquisition and stitching time for one complete stitched image was approximately 1.5 min. Image preprocessing and boundary extraction were performed as described above. Briefly, the hydrogel region was divided into 80 bins along the x-axis, each corresponding to a 0.1 × 4 mm region. The protocell-associated fluorescence intensity within each bin was summed to obtain a spatial fluorescence profile, which was then smoothed, normalized and binarized using a threshold of 0.2. Boundary positions were determined from the peak of the differentiated binary profile calculated using the MATLAB gradient function.

**Full-adder logic computing in protocell collective responses.** A GOx/RITC-BSA loaded proteinosomes solution (5 × 10^5^ pcells/mL, 120 μL) was mixed with agarose solution (0.2 wt%, 80 μL). A total of 200 μL of protocell hydrogel (3 × 10^5^ protocells/mL) was then loaded into a rectangular chamber (L/W/H: 20/5/2 mm), which was cut from an acrylic plate and adhered to a coverslip at the bottom. The entire setup was placed in a confocal dish filled with water to prevent dehydration of the hydrogel. A fixed glucose solution (500 mM, 2 μL) was added to both lateral sides of the hydrogel. A gradient of different reducing agents was introduced at the center of the hydrogel’s long axis, according to eight distinct conditions. The reducing agent inputs consisted of TCEP, DTT, and DTE at the following concentration combinations (TCEP/DTT/DTE: 0/0/0; 0/0/50; 0/25/0; 0/25/50; 50/0/0; 50/0/50; 50/25/0; 50/25/50 mM), with a total volume of 2 μL added for each agent (resulting in a combined volume of 6 μL for triple-agent conditions). Each condition was repeated three times. For each individual repeat, TCEP, DTT, or DTE stock solutions were freshly prepared before use, and the pH of each reductant stock solution was adjusted to 8.5 prior to addition. Replicate measurements for the same condition were performed on the same day. After 12 hours of incubation, wide-field images of the hydrogel were captured for each condition using confocal microscopy. For the 20 × 5 mm hydrogel region, the full area was acquired as an 18 × 5 stitched mosaic, and the acquisition and stitching time for one complete stitched image was approximately 3.2 min. Image preprocessing and boundary extraction were performed as described above. Briefly, the hydrogel region was divided into 200 bins along the x-axis, each corresponding to a 0.1 × 5 mm region. The protocell-associated fluorescence intensity within each bin was summed to obtain a spatial fluorescence profile, which was then smoothed, normalized and binarized using a threshold of 0.2. Boundary positions were determined from the peak of the differentiated binary profile calculated using the MATLAB gradient function.

**Computation simulations of the protocell system.** The simulation method followed the approach described previously^[1]^. In the simulation, the following assumptions were made: (i) the protocells were monodisperse and their distribution was homogeneous; (ii) there was no transfer and no leakage of BSA in protocells unless disulfide bonds were destroyed; (iii) the concentration of proteins in the protocells was uniform; (iv) small molecule diffusion is not affected by the proteinosome membrane, and (v) the influence of the diffusion of GOx on the rate of hydrogen peroxide production can be neglected.

The substance changes in enzymatic reaction and redox reactions in this system can be described by the following reaction rate equations:

$$\begin{aligned} \frac{\partial C\left( S-S \right)}{\partial t} = -k_{1}C\left( TCEP \right)C\left( S-S \right)\#\left( 1 \right) \end{aligned}$$

$$\begin{aligned} \frac{\partial C\left( TCEP \right)}{\partial t} = -k_{1}C\left( TCEP \right)C\left( S-S \right)-k_{3}C\left( H_{2}O_{2} \right)C\left( TCEP \right)\#\left( 2 \right) \end{aligned}$$

$$\begin{aligned} \frac{\partial C\left( H_{2}O_{2} \right)}{\partial t} = k_{2}C\left( Glucose \right)C\left( O_{2} \right)-k_{3}C\left( H_{2}O_{2} \right)C\left( TCEP \right)\#\left( 3 \right) \end{aligned}$$

where C(O_2_) was 267 μM; *k_1_*=0.83 M^-1^ s^-1^, was rate constant for TCEP reduction of disulfide bonds; *k_2_*=15.1 M^-1^ s^-1^, was GOx-mediated rate of glucose conversion to gluconic acid and hydrogen peroxide; *k_3_*=5.6 x 10^8^ M^-1^ s^-1^, was rate constant for redox reaction between TCEP and hydrogen peroxide.

The distribution of protocells (average size: 25 μm; concentration: 3 x 10^5^ protocells/mL) in rectangle chamber (L/W/H : 8/4/2.5 mm) was assumed homogeneous along 3 dimensions (x, y, z). Every protocell was positioned in the central region of a 150 x 150 x 150 μm cubic space. Therefore, the diffusion process consists of two parts, one is the diffusion of TCEP, H_2_O_2_, glucose and BSA between the cubes; the other is the diffusion of AGx and BSA from the protocells within the cubes.

The diffusion coefficient of TCEP, H_2_O_2_, glucose, BSA in the hydrogel were estimated as 6 x 10^-6^ cm^2^ s^-1^, 6.75 x 10^-6^ cm^2^ s^-1^, 6 x 10^-6^ cm^2^ s^-1^, 6.5 x 10^-7^ cm^2^ s^-1^.

The diffusion of each species between the cubes was modeled using Fick's law in 3D, simplified for a 1D diffusion process along the x-axis:

$$\begin{aligned} \frac{\partial C\left( S \right)}{\partial t}=D\left( S \right)\frac{\partial^{2}C\left( S \right)}{\partial x^{2}}\#\left( 4 \right) \end{aligned}$$

The finite difference method was used to simplify equation (4):

$$\begin{aligned} \frac{\Delta C\left( S \right)}{\Delta t}=\frac{D\left( S \right)\left( {C\left( S \right)}_{x+1}+{C\left( S \right)}_{x-1}-2\times{C\left( S \right)}_{x} \right)}{L^{2}}\#\left( 5 \right) \end{aligned}$$

$$\begin{aligned} F_{0} = \frac{D\left( S \right)\times\Delta t}{L^{2}} \leq0.25\#\left( 6 \right) \end{aligned}$$

where *S* represented TCEP, H_2_O_2_, glucose and BSA outside the protocells; *L* was set as 150 μm, representing the distance of adjacent cubes; *Δt* was set less to than 9.375s, such that the stability factor (*F_0_*) is less than 0.25 to ensure the stability of simulation.

Within the cubes, as disulfide bonds got reduced, BSA diffused out of the protocell along 3 dimensions in a cube. The 3D diffusion profile of BSA can also be simplified as below:

$$\begin{aligned} \frac{\Delta C\left( BSA \right)_{in}}{\Delta t} = -D^{'}\left( BSA \right)\left( 3\times\frac{2\times\left( C\left( BSA \right)_{in}-C\left( BSA \right)_{out} \right)}{d^{2}} \right)\#\left( 7 \right) \end{aligned}$$

$$\begin{aligned} D^{'}\left( BSA \right) = -0.074+\frac{0.986}{1+\left( \frac{C\left( S-S \right)}{0.074} \right)^{0.059}}\#\left( 8 \right) \end{aligned}$$

where *D′(BSA)* is the relative diffusion coefficient of the protein BSA, related to disulfide crosslinking on the membrane; *d*≈43.75 *μ*mdenotes the effective diffusion length scale, estimated as the distance between the center of protocell (*r* ≈ 12.5 μm) and the outer mid-point along the x-axis.

**2. Supplementary figures**

**Figure S1.** (**A-B**) Confocal fluorescence images of GOx/RITC-BSA-containing proteinosomes (**A**), and corresponding size distributions (**B**). Scale bars: 200 μm.

**Figure S2.** Single-protocell fluorescence analysis of GOx/RITC-BSA-loaded proteinosomes under homogeneous reducing conditions. (**A**) Time-lapse fluorescence microscopy images of GOx/RITC-BSA-loaded proteinosomes (7.2 × 10^5^ protocells/mL) upon exposure to DTT, DTE or TCEP (final concentration: 0.25 mM). Scale bars: 100 μm. (**B-D**) Time-dependent fluorescence intensity changes of individual protocells upon exposure to DTT (**B**), DTE (**C**) or TCEP (**D**). The fluorescence intensity of each protocell was normalized to its initial value. (**E**) Box plot of the time required for the fluorescence intensity of individual protocells to decrease to 20% of the initial value (t_20%_) under DTT, DTE and TCEP treatment. For each condition, n = 20 individual protocells.

**Figure S3.** (**A-C**) Time-dependent plots of the change of fluorescence intensity of the GOx/RITC-BSA-loaded proteinosomes (7.2 x 10^5^ protocells/mL) upon exposure to different ratios of reductant concentrations. The binary mixtures tested are **(A)** DTT/TCEP, **(B)** DTT/DTE, and **(C)** DTE/TCEP. For all groups, the total concentration was maintained at 0.25 mM, with molar ratios of 4:0, 3:1, 2:2, 1:3, and 0:4 (corresponding to concentrations of 0.25/0, 0.188/0.062, 0.125/0.125, 0.062/0.188, and 0/0.25 mM, respectively). (**C-E**) Comparison of decay times (t_20%_) across various binary reductant mixtures, extracted from (**A-C**). t_20%_ denotes the time required for the fluorescence intensity of proteinosomes (7.2 x 10^5^ protocells/mL) to decrease to 20% of the initial value. Data are shown for (**C**) DTT/TCEP, (**D**) DTT/DTE, and (**E**) DTE/TCEP mixtures.

**Figure S4.** (**A**) Schematic of membrane disassembly and fluorescent cargo release in a GOx-containing protocell model upon reductant exposure. Glucose-driven H_2_O_2_ production by GOx inhibits reductant-induced membrane rupture and cargo leakage. (**B**) Time-dependent plots of the change of fluorescence intensity of the GOx/RITC-BSA-loaded proteinosomes (7.2 x 10^5^ protocells/mL) upon exposure to 0.25 mM DTT, DTE, or TCEP, alone or with 0.25 mM glucose.

**Figure S5.** (**A**) Confocal fluorescence microscopy image of a hydrogel-immobilized homogeneous GOx/RITC-BSA-loaded proteinosomes (3 x 10^5^ protocells/mL). Scale bar: 2 mm. (**B**) Population-averaged fluorescence intensity profile extracted from image (**A**) along the x axis. For this analysis, the hydrogel image was divided into consecutive vertical bins with a width of 30 μm, and the fluorescence intensity within each bin was averaged to generate the spatial fluorescence profile.

**Figure S6.** (**A-B**) 2D plots of normalized fluorescence intensity (colour side bar) inside the protocells (3 x 10^5^ protocells/mL) recorded at different times (t) and positions along the *x* axis after injection of TCEP concentration (2.5 μL, 500 mM, left), in the presence (**A**) or absence (**B**) of glucose gradient (2.5 μL, 500 mM, right). (**C**) Time-dependent boundary positions extracted from three independently prepared proteinosome-containing hydrogel samples (I, II and III) under the same gradient condition, showing the reproducibility of boundary displacement. (**D**) Box plots of boundary positions after the boundary reached a stable state. For each independently prepared sample, boundary positions were extracted from 60 min to 12 h at 6 min intervals, resulting in 110 data points per sample. The stable boundary positions were 4.28 ± 0.13 mm for sample I, 4.28 ± 0.08 mm for sample II and 4.11 ± 0.05 mm for sample III.

**Figure S7.** (**A-F**) Plots of boundary positions of protocell responses against different TCEP concentrations (31.25-500 mM, 2 μL, left) with varying protocell densities: 0.75 x 10^5^ protocells/mL (**A**); 1.50 x 10^5^ protocells/mL (**B**); 2.25 x 10^5^ protocells/mL (**C**); 3.00 x 10^5^ protocells/mL (**D**); 4.50 x 10^5^ protocells/mL (**E**); 6.00 x 10^5^ protocells/mL (**F**). Fixed glucose gradients (500 mM, 2 μL) were applied on right side. (**G**) Heatmap of boundary positions of protocell responses against different TCEP concentrations and protocell densities.

**Figure S8.** (**A-C**) 2D plots of simulated TCEP (**A**), H_2_O_2_ (**B**) and glucose (**C**) concentrations in hydrogel along x axis. Simulation was conducted in the protocell hydrogel (3 x 10^5^ protocells/mL) upon opposing gradients of TCEP (2.5 μL, 500 mM, left) and glucose (2.5 μL, 500 mM, right).

**Figure S9.** (**A**) Plots of the time required for the BSA concentration to decay to 20% (t_20%_) inside the protocell (3 x 10^5^ protocells/mL) against the position of the protocell along the chamber upon opposing gradients of reductant (1 μL, 500 mM, left) and glucose (1 μL, 500 mM, right), under artificially changed diffusion coefficients of reductant (*D_reductant_*). (**B**) Plots of the time required for the BSA concentration to decay to 20% (t_20%_) inside the protocell (3 x 10^5^ protocells/mL) against the position of the protocell along the chamber upon opposing gradients of reductant (1 μL, 50 mM, left) and glucose (1 μL, 500 mM, right), under artificially changed reaction rate (*k_1_*) between reductant and disulfide bonds within proteinosomes.

**Figure S10.** Bar chart of boundary positions extracted from simulated BSA concentrations in hydrogel-immobilized, homogeneous GOx/RITC-BSA-loaded proteinosomes (3 x 10^5^ protocells/mL), upon opposing gradients of glucose (1 μL, 500 mM, right) and reductant (1 μL, 10/100/250/500 mM, left). Simulations were conducted under artificially changed diffusion coefficient of reductant (*D_H_*/*D_L_*, 7/6 x 10^-6^ cm^2^/s) and reaction rate (*k_1H_*/*k_1L_*, 1/0.5 M^-1^s^-1^) between reductant and disulfide bonds within proteinosomes.

**Figure S11.** (**A-C**) Confocal fluorescence microscopy images of hydrogel-immobilized, homogeneous GOx/RITC-BSA-loaded proteinosomes (3 x 10^5^ protocells/mL) recorded 12 h, upon opposing gradients of reductant (1 μL, 100 mM, left) and glucose (1 μL, 500 mM, right). Images are shown for (**A**) TCEP, (**B**) DTE, and (**C**) DTT. Scale bars: 1 mm.

**Figure S12.** Bar chart of boundary positions extracted from the confocal fluorescence microscopy images of hydrogel-immobilized, homogeneous GOx/RITC-BSA-loaded proteinosomes (3 x 10^5^ protocells/mL) recorded 12 h, upon opposing gradients of glucose (1 μL, 500 mM, right) and DTT, DTE, or TCEP reductant (1 μL, 50/100/150 mM, left). Each condition was performed in triplicate (n = 3), with individual data points shown in red and bars representing mean ± s.d. Boundary positions were 3.12 ± 0.06, 3.42 ± 0.06, and 3.58 ± 0.06 mm for DTT at 50, 100, and 150 mM; 2.92 ± 0.06, 3.18 ± 0.06, and 3.42 ± 0.06 mm for DTE at 50, 100, and 150 mM; and 2.55 ± 0.10, 2.88 ± 0.06, and 3.18 ± 0.06 mm for TCEP at 50, 100, and 150 mM, respectively.

**Figure S13.** (**A–D**) Confocal fluorescence microscopy images of homogeneous proteinosome-containing hydrogels (3 × 10^5^ protocells mL^-1^) recorded over 12 h after the addition of reductant inputs from the left side and glucose from the right side. The reductant inputs were 1 μL of TCEP/DTE/DTT at concentrations of 50/0/0 mM (**A**), 0/50/0 mM (**B**), 0/0/50 mM (**C**), and 50/50/50 mM (**D**), respectively. Glucose was added from the right side at 500 mM in a volume of 1 μL. Scale bars: 1 mm. (**E**) Normalized fluorescence intensity profiles extracted along the x axis from the corresponding microscopy images in (**A–D**). Data are presented as mean ± s.d. from three independent experiments (n = 3).

**Figure S14.** (**A-D**) Boundary positions extracted from protocell-containing hydrogels (3 × 10^5^ protocells mL^-1^) under mixed TCEP/DTT inputs. In each panel, the TCEP concentration was fixed at 31.25 mM (**A**), 62.5 mM (**B**), 125 mM (**C**), or 250 mM (**D**), while the DTT concentration was varied from 0 to 250 mM. For each condition, the TCEP/DTT mixture (2/2 μL) was introduced from the left side of the hydrogel, and glucose solution (500 mM, 2 μL) was introduced from the right side. (**E**) Heatmap of boundary positions of protocell responses against different TCEP and DTT concentrations.

**Figure S15.** (**A-D**) Confocal fluorescence microscopy images of hydrogel-immobilized, homogeneous GOx/RITC-BSA-loaded proteinosomes (3 x 10^5^ protocells/mL) recorded 12 h, under different TCEP/DTT inputs: 0/0 (**A**); 0/1 (**B**); 1/0 (**C**); 1/1 (**D**). For each combination (TCEP/DTT in mM [logical value]: 0/0 [0/0], 0/31.25 [0/1], 62.5/0 [1/0], 62.5/31.25 [1/1]), a TCEP/DTT gradient was applied to the left (2/2 μL) and a fixed glucose gradient (500 mM, 2 μL) to the right. Scale bars: 2 mm. (**E-H**) Plots of the normalized fluorescence intensity extracted from the corresponding microscopy images (**A-D**) along x axis. For spatial profiling, the 8 × 4 mm hydrogel region was divided into 80 bins along the x-axis, with each bin corresponding to a 0.1 × 4 mm region. The protocell-associated fluorescence intensity within each bin was summed to generate the spatial fluorescence profile, which was then normalized for boundary analysis. (**I-L**) Corresponding binary plots of the normalized intensity by applying a threshold of 0.2 to the data from (**E-H**) through triplicate experiments (n = 3), respectively. (**M-P**) Corresponding plots of the gradient, calculated from the binary normalized intensity data in (**I-L**), respectively.

**Figure S16.** (**A, E, I**) Confocal fluorescence microscopy images of hydrogel-immobilized, homogeneous GOx/RITC-BSA-loaded proteinosomes (3 x 10^5^ protocells/mL) recorded 12 h, under different TCEP/DTT/DTE inputs: 1/0/0 (**A**); 0/1/0 (**E**); 0/0/1 (**I**). For each combination (TCEP/DTT/DTE in mM [logical value]: 50/0/0 [1/0/0], 0/25/0 [0/1/0], 0/0/50 [0/0/1]), a TCEP/DTT/DTE gradient was applied to the center of the hydrogel (2/2/2 μL) and fixed glucose gradients (500 mM, 2 μL) were applied on both sides. Scale bars: 2 mm. (**B, F, J**) Plots of the normalized fluorescence intensity extracted from the corresponding microscopy images (**A, E, I**) along x axis. For spatial profiling, the 20 x 5 mm hydrogel region was divided into 200 bins along the x-axis, with each bin corresponding to a 0.1 × 5 mm region. The protocell-associated fluorescence intensity within each bin was summed to generate the spatial fluorescence profile, which was then normalized for boundary analysis. (**C, G, K**) Corresponding binary plots of the normalized intensity by applying a threshold of 0.2 to the data from (**B, F, J**) through triplicate experiments (n = 3), respectively. (**D, H, L**) Corresponding plots of the gradient, calculated from the binary normalized intensity data in (**C, G, K**), respectively.

**Figure S17.** (**A, E, I**) Confocal fluorescence microscopy images of hydrogel-immobilized, homogeneous GOx/RITC-BSA-loaded proteinosomes (3 x 10^5^ protocells/mL) recorded 12 h, under different TCEP/DTT/DTE inputs: 1/1/0 (**A**); 1/0/1 (**E**); 0/1/1 (**I**). For each combination (TCEP/DTT/DTE in mM [logical value]: 50/25/0 [1/1/0], 50/0/50 [1/0/1], 0/25/50 [0/1/1]), a TCEP/DTT/DTE gradient was applied to the center of the hydrogel (2/2/2 μL) and fixed glucose gradients (500 mM, 2 μL) were applied on both sides. Scale bars: 2 mm. (**B, F, J**) Plots of the normalized fluorescence intensity extracted from the corresponding microscopy images (**A, E, I**) along x axis. For spatial profiling, the 20 x 5 mm hydrogel region was divided into 200 bins along the x-axis, with each bin corresponding to a 0.1 × 5 mm region. The protocell-associated fluorescence intensity within each bin was summed to generate the spatial fluorescence profile, which was then normalized for boundary analysis. (**C, G, K**) Corresponding binary plots of the normalized intensity by applying a threshold of 0.2 to the data from (**B, F, J**) through triplicate experiments (n = 3), respectively. (**D, H, L**) Corresponding plots of the gradient, calculated from the binary normalized intensity data in (**C, G, K**), respectively.

**Figure S18.** (**A, E**) Confocal fluorescence microscopy images of hydrogel-immobilized, homogeneous GOx/RITC-BSA-loaded proteinosomes (3 x 10^5^ protocells/mL) recorded 12 h, under different TCEP/DTT/DTE inputs: 1/1/1 (**A**); 0/0/0 (**E**). For each combination (TCEP/DTT/DTE in mM [logical value]: 50/25/50 [1/1/1], 0/0/0 [0/0/0]), a TCEP/DTT/DTE gradient was applied to the center of the hydrogel (2/2/2 μL) and fixed glucose gradients (500 mM, 2 μL) were applied on both sides. Scale bars: 2 mm. (**B, F**) Plots of the normalized fluorescence intensity extracted from the corresponding microscopy images (**A, E**) along x axis. For spatial profiling, the 20 x 5 mm hydrogel region was divided into 200 bins along the x-axis, with each bin corresponding to a 0.1 × 5 mm region. The protocell-associated fluorescence intensity within each bin was summed to generate the spatial fluorescence profile, which was then normalized for boundary analysis. (**C, G**) Corresponding binary plots of the normalized intensity by applying a threshold of 0.2 to the data from (**B, F**) through triplicate experiments (n = 3), respectively. (**D, H**) Corresponding plots of the gradient, calculated from the binary normalized intensity data in (**C, G**), respectively.

**Figure S19.** (**A-B**) Bar charts of boundary positions of protocell responses (protocell density: 3 × 10⁵ /mL) in the Sum-defining regions (Y_5_ and Y_7_, where signal in either yields Y_n_=1, **A**) and the Carry-out-defining regions (Y_15_ and Y_16_, where signal in either yields Y_m_=1, **B**), under all eight input conditions (Inputs: TCEP/DTT/DTE; “1” = 50/25/50 mM, 2 μL, central; “0” = absent). TCEP/DTT/DTE gradient was applied to the center of the hydrogel and fixed glucose gradients (500 mM, 2 μL) were applied on both sides. For the input conditions 000, 001, 010, 011, 100, 101, 110 and 111, the boundary positions in the Sum-defining regions were 0 ± 0, 6.38 ± 0.15, 6.28 ± 0.21, 5.28 ± 0.06, 6.68 ± 0.25, 5.28 ± 0.06, 5.52 ± 0.15 and 4.38 ± 0.32 mm, respectively. The corresponding boundary positions in the Carry-out-defining regions were 0 ± 0, 13.32 ± 0.21, 13.52 ± 0.06, 14.75 ± 0.10, 13.28 ± 0.15, 14.68 ± 0.06, 14.42 ± 0.06 and 15.35 ± 0.10 mm, respectively. Data are presented as mean ± s.d. with individual data points shown; n = 3 independent repeats.

**Reference**

[1]	S. Wu, I. Myrgorodska, M. Li, P. Xu, S. Mann, L. Tian, Protocell Survival Networks in Spatially Modulated Chemical Gradients.  *J Am Chem Soc* **147** (2025): 44804–44816.
